# Suboptimal Weight Loss 13 Years After Roux-en-Y Gastric Bypass: Is Hedonic Hunger, Eating Behaviour and Food Reward to Blame?

**DOI:** 10.1007/s11695-022-06075-z

**Published:** 2022-05-04

**Authors:** Siren Nymo, Oda Børresen Skjølsvold, Marthe Aukan, Graham Finlayson, Hallvard Græslie, Ronald Mårvik, Bård Kulseng, Jorunn Sandvik, Catia Martins

**Affiliations:** 1 Obesity Research Group, Department of Clinical and Molecular Medicine, Faculty of Medicine and Health Sciences, Norwegian University of Science and Technology (NTNU), Prinsesse Kristinas gate 5, 7030 Forsyningssenteret, Trondheim, Norway; 2 Centre for Obesity and Innovation (ObeCe), Clinic of Surgery, St. Olav University Hospital, Trondheim, Norway; 3 Clinic of Surgery, Namsos Hospital, Nord-Trøndelag Hospital Trust, Namsos, Norway; 4 School of Psychology, University of Leeds, Leeds, UK; 5 Clinic of Surgery, Ålesund Hospital, Møre- og Romsdal Hospital Trust, Ålesund, Norway; 6 Department of Nutrition Sciences, University of Alabama at Birmingham, Birmingham, AL USA

**Keywords:** Hedonic hunger, Food preferences, Food reward, Weight loss, Bariatric surgery

## Abstract

**Purpose:**

Suboptimal weight loss (SWL) and weight regain (WR) following bariatric surgery are common. The exact reasons for this phenomenon remain to be fully elucidated. To compare hedonic hunger, food preferences, food reward and eating behaviour traits between participants with SWL and optimal weight loss (OWL) 13 years after Roux-en-Y gastric bypass (RYGB).

**Materials and Method:**

Cross-sectional case control study where participants experiencing SWL or OWL (< or ≥ 50% of excess weight, respectively) post-RYGB were compared to a non-surgical control group matched for pre-operative body mass index. Hedonic hunger (Power of Food Scale), implicit and explicit liking and wanting for high-fat and low-fat savoury and sweet food (Leeds Food Preference Questionnaire) and eating behaviour (Dutch Eating Behavior Questionnaire, Three-Factor Eating Questionnaire and the Food Cravings Questionnaires State and Trait-reduced) were assessed.

**Results:**

In total, 75 participants were recruited from the bariatric surgery observation study (BAROBS). Disinhibition, hunger, emotional, external and restrained eating, frequency of cravings and hedonic hunger were lower in the OWL, compared with the SWL and/or control groups. Implicit wanting and explicit liking and wanting for high-fat savoury and high-fat sweet food were lower, and implicit wanting for low-fat savoury food higher, in the OWL, compared with the SWL and/or control groups.

**Conclusion:**

SWL 13 years after RYGB is associated with dysfunctional eating behaviours, increased preference and reward for high-fat food and increased hedonic hunger. Future longitudinal studies are needed to establish the cause-effect relationship between these variables.

**Graphical abstract:**

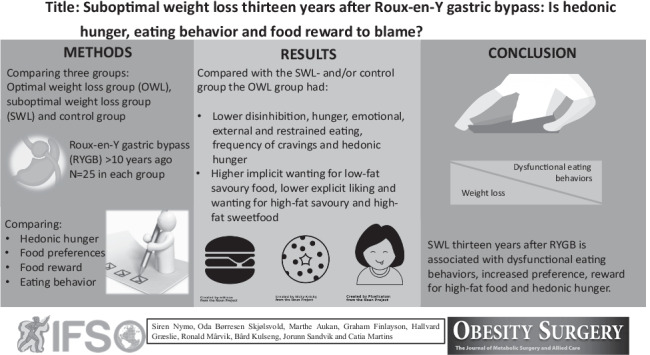

**Supplementary Information:**

The online version contains supplementary material available at 10.1007/s11695-022-06075-z.

## Introduction

Bariatric surgery is the most effective treatment for severe obesity [1–3], with a total weight loss (TWL) of approximately 40% at 2-year follow-up [4]. Roux-en-Y gastric bypass (RYGB) was, until recently, one of the most common bariatric procedures performed worldwide [5, 6]. Unfortunately, approximately 30% of patients experience suboptimal weight loss (SWL) and/or weight regain (WR) post-RYGB [7–9]. Significant WR is defined as ≥ 15% regain from nadir, and a longer interval after RYGB has been associated with weight regain (as been reported in 17% of the patients after 2-year follow-up) [10]. Unfortunately, a clear-cut definition of post-bariatric surgery WR and WL failure is missing [11, 12].

The mechanisms by which RYGB induces weight loss are not fully understood. However, the anatomical exclusion of the foregut, and subsequent upregulation of the secretion of glucagon-like peptide-1 and peptide YY, known to promote satiety and reduce food intake, is likely to be involved [13]. The aetiology of SWL and WR post-bariatric surgery remains to be fully elucidated [7, 14–21]. The available evidence suggests that hedonic hunger [22, 23], food preferences [18] and eating behaviour [17, 19] also modulate WL outcomes post-RYGB and might contribute to SWL and WR. Dysfunctional eating, defined as loss of control over eating, eating for emotional reasons, exerting strict control or eating without actually being hungry [24], is often seen in candidates for bariatric surgery [24, 25], and changes in this behaviour have been shown to modulate WL outcomes post-bariatric surgery [26]. RYGB is associated with an overall reduction in hedonic hunger and the drive to consume palatable foods [22, 23], and an increased desire for less energy dense foods [22, 27, 28]. More importantly, a positive association has been found between explicit liking for high-fat sweet (HFSW) food and WR post-RYGB [29].

Despite the evidence previously described, little is known regarding how inter-individual differences in hedonic hunger, food preferences and reward and eating behaviour post-RYGB contribute to SWL and WR in the long term. Therefore, the main aim of this study was to compare food preferences and reward, hedonic hunger and eating behaviour traits between participants with SWL and optimal WL (OWL) 13 years post-RYGB, and a non-surgical control group matched for the pre-operative body mass index (BMI) of the bariatric groups. A secondary aim was to explore the association between food preferences and reward, hedonic hunger and eating behaviour traits and percent of total WL (%TWL) and excess WL (%EWL) 13 years post-RYGB.

## Materials and Method

### Study Design

This is a cross-sectional case control study. Participants who had undergone RYGB between 2003 and 2009 (average 13 years earlier) were invited to participate in this study and were compared to a control group who had not undergone RYGB and presented with a similar pre-operative BMI. SWL was defined as < 50% of EWL and OWL as > 50% of EWL [30, 31]. The RYGB procedure was performed laparoscopically according to the Lönroth technique, with a pouch of 15–30 ml, biliopancreatic limb of 40–60 cm, antegastric, antecolic Roux limb of 100 cm if preoperative BMI ≤ 40 kg/m^2^, and 150 cm if preoperative BMI > 50 kg/m^2^, and linear stapled gastrojejunostomy and jejunojejunostomy. The mesenteric defects were not closed at that time [32]. The main indication for surgery in this population was weight loss.

### Participants

Participants were recruited from the Bariatric Surgery Observation Study (BAROBS), a follow-up health survey in three local hospitals in the Central Norway Health Region. A total of 936 males and females were invited (28–75 years), operated between 2003 and 2009. The pre-operative control group included participants enrolled in the DISGAP-study (‘Effect of **DI**et versus **S**leeve **G**astrectomy and gastric bypass on **AP**petite’), who were on the waiting list for obesity treatment. Participants who had revisional bariatric surgery, eating disorders, were pregnant or breastfeeding, or were taking medications known to affect body weight or appetite (such as thyroid hormones and weight loss drugs) were excluded from the study.

Both the BAROBS and DISGAP studies were approved by the local ethics committee (REK 2017/1828-21and 2019/252, respectively). Additionally, the DISGAP study was registered in Clinical trials (NCT04051190). All participants provided written informed consent in line with the Helsinki Declaration, before entering the studies.

### Measurements

All measurements were taken in average 13 years after surgery. Anthropometric measurements were taken in the fasting state (12 h), and after that participants answered the Leeds Food Preference Questionnaire (LFPQ) [33]. A liquid test meal was then offered, and participants asked to drink the shake over a 20-min period to avoid dumping symptoms. The test meal consisted of 200 ml of Diben shake (Fresenius Kabi, Fredrikstad, Norway) (Nutritional composition per 200 ml: 300 kcal, 15 g protein, 14 g fat and 26 g carbohydrates). Following this, participants answered the LFPQ again and filled out a battery of questionnaires. Detailed information about all measurements is described below.

### Anthopometrics

Height was measured with a Seca 217 stadiometer (Seca, Hamburg, Germany) to the nearest 0.5 cm and weight was measured with a Seca 877 digital flat scale (Seca, Hamburg, Germany) to the nearest 0.1 kg. Body composition was assessed with air-displacement plethysmography (BodPod, Cosmed, Concord, CA, USA). The Brozeq equation was used to determine fat mass (FM) [34].

Pre-operative weight was the weight closest to the time of surgery. Ideal weight was the weight corresponding to a BMI of 25 kg/m^2^. %EWL, %WR and %TWL were estimated using standard equations [4, 12, 35].

### Eating Behaviour

Eating behaviour traits were assessed after the test meal with four different questionnaires: Three-Factor Eating Questionnaire (TFEQ) [36], Dutch Eating Behavior Questionnaire (DEBQ) [37] and Food Cravings Questionnaires Trait-reduced and State (FCQ-T-r and FCQ-S) [38, 39]. TFEQ measures three different dimensions: restraint, disinhibition and hunger. TFEQ was developed for application in obesity research and has been found to be suitable in identifying subjects with eating behaviours that are associated with higher BMI [40, 41]. DEBQ measures cognitive restrained eating, external eating and emotional eating. Emotional eating can be divided into two sub-categories: diffuse emotions and clearly labelled emotions. FCQ-T-r measures frequency of cravings, while FCQ-S measures intensity of cravings [42]. The FCQ-S has been validated for use in both clinical and nonclinical populations [43, 44].

### Hedonic Hunger

Power of Food Scale (PFS) was used to measure hedonic hunger in fed state. It has three different categories: food tasted, food present and food available, in addition to an aggregated score for all the categories combined [45]. PFS has been found to have good reliability for measuring hedonic hunger in both the general population and in individuals with obesity [45, 46].

### Liking and Wanting for Food

LFPQ was used to measure ‘liking’ and ‘wanting’ for food, before and after the test meal [47]. Implicit measures of wanting and explicit measures of liking and wanting were measured by completing computer tasks lasting approximately 10 min. Food preference and food reward were assessed by pictures of common food items adjusted to the Norwegian diet. In addition, the pictures were modified when necessary (for participants with allergies and/or intolerances to the food pictures presented). The food pictures have two dimensions: fat (high or low) and taste (sweet or savoury) and can be divided into four categories: high fat savoury (HFSA), low fat savoury (LFSA), high fat sweet (HFSW) or low fat sweet (LFSW) [33]. LFPQ has been modified and adapted several times since it was created by Finlayson et al. (2007) [47, 48].

### Statistical Analysis

Statistical analysis was performed using IBM SPSS Statistics 26 (SPSS In., Chicago, IL, USA), and data presented as mean ± SEM, unless stated otherwise. Statistical significance was assumed at *P* < 0.05. Differences between groups (SWL, OWL and control) were assessed with one-way ANCOVA after adjusting for age and preoperative BMI. Bonferroni correction was used for multiple pairwise comparisons. Differences between groups for liking and wanting for food were assessed with Kruskal–Wallis followed by Mann–Whitney *U*-test, as these variables were not normally distributed. Correlation between scores from questionnaires and liking and wanting from LFPQ, and %EWL, %TWL and %WR, in the bariatric groups, was performed with Pearson or Spearman correlation, depending on the normality of the data.

## Results

Participants’ characteristics are reported in Table 1. Seventy-five participants (79% women) participated in this study, 25 participants in each group, with a similar sex distribution. The control group was younger than both the SWL and OWL groups (*P* < 0.01 and *P* < 0.05, respectively). The OWL group had a lower BMI, weight and %FM compared with both the SWL and control groups (*P* < 0.01 for all). There were no significant differences in BMI between the SWL group and control group. Despite no significant differences in pre-operative BMI between the control and the bariatric groups combined, the SWL group presented with a higher preoperative BMI compared with both control and OWL groups (*P* < 0.01). The SWL group also presented with higher nadir weight, and %WR (*P* < 0.01 for both) and lower %TWL and %EWL (*P* < 0.01 for both) compared with the OWL group. Nineteen participants (76%) in the SWL group also had > 15% WR. Only two (8%) participants in each group (SWL and OWL) were on medication for type 2 diabetes.

**Table 1:** Baseline characteristics of participants

	SWL (*n* = 25)	OWL (*n* = 25)	Control (*n* = 25)
Age (years)	50.5 ± 1.9^a^	52.2 ± 1.9^b^	44.4 ± 1.9^ab^
Years since surgery	13 ± 0.3	13 ± 0.2	-
Female *n* (%)	20 (80)	23 (92)	16 (64)
Weight (kg)	124.9 ± 3.3^b^	75.3 ± 3.3^bc^	123.7 ± 3.3^c^
BMI (kg/m^2^)	42.9 ± 0.8^b^	27.0 ± 0.8^bc^	41.8 ± 0.8^c^
Pre-operative BMI (kg/m^2)^	46.3 ± 0.8^bc^	41.4 ± 0.8^b^	41.8 ± 0.8^c^
Pre-operative weight (kg/m^2)^	135.8 ± 3.6^b^	116.9 ± 3.6^b^	-
Nadir (kg)	98.0 ± 2.6^b^	72.1 ± 2.6^b^	-
%TWL	7.7 ± 1.9^b^	35.2 ± 1.9^b^	-
%EWL	16.6 ± 4.1^b^	87.3 ± 4.1^b^	-
%WR	19.7 ± 2.6^b^	4.5 ± 2.6^b^	-

Data presented as estimated marginal means ± SEM. Means with same superscript letters are significantly different ^a^*P* < 0.05, ^b,c^*P* < 0.01. *SWL*, suboptimal weight loss; *OWL*, optimal weight loss; *BMI*, body mass index; *%TWL*, percent total weight loss; *%EWL*, percent excess weight loss; *%WR*, percent weight regain

Results for eating behaviour traits are presented in Table 2. The OWL group presented with a lower score for disinhibition and hunger (TFEQ) compared with both the SWL and control groups (*P* < 0.01 for all). Dietary restraint score was also lower in the OWL compared with the control group (*P* < 0.05). Emotional eating, external eating and diffuse and clearly labelled emotions from DEBQ were lower in the OWL compared with both the SWL and control groups (*P* < 0.05 and *P* < 0.01). Restrained eating from DEBQ was also lower in the OWL compared with the control group only (*P* < 0.01). Frequency of cravings (FCQ-T-r) was higher in the SWL and control groups compared with the OWL group (*P* < 0.01 for both), but there were no differences among groups for intensity of cravings (FCQ-S). Significant differences among groups were seen in all four categories of PFS. Aggregated score, food present and food available were lower in the OWL group compared with both the SWL (*P* < 0.05) and control groups (*P* < 0.01). The score for food tasted was also lower in the OWL compared with the control group only (*P* < 0.05).

**Table 2:** Eating behaviour traits and hedonic hunger in the different study groups

	SWL (*n* = 25)	OWL (*n* = 25)	Control (*n* = 25)
TFEQ			
Dietary restraint	9.2 ± 0.9	8.2 ± 0.9^a^	11.3 ± 0.8^a^
Disinhibition	7.8 ± 0.7^b^	3.8 ± 0.7^bc^	9.0 ± 0.7^c^
Hunger	6.4 ± 0.7^b^	3.0 ± 0.6^bc^	6.0 ± 0.6^c^
DEBQ			
Emotional eating	3.1 ± 0.2^b^	1.9 ± 0.2^ab^	2.6 ± 0.2^a^
Restrained eating	2.8 ± 0.1	2.5 ± 0.1^b^	3.0 ± 0.1^b^
External eating	3.1 ± 0.1^a^	2.7 ± 0.1^ab^	3.3 ± 0.1^b^
Diffuse emotions	3.2 ± 0.2^b^	2.1 ± 0.2^bc^	2.9 ± 0.2^c^
Clearly labelled emotions	3.0 ± 0.2^b^	1.8 ± 0.2^ba^	2.5 ± 0.2^a^
FCQ			
FCQ-S	43.7 ± 3.6	33.2 ± 3.4	39.8 ± 3.1
FCQ-T-r	38.4 ± 2.8^b^	24.1 ± 2.7^bc^	40.9 ± 2.8^c^
PFS			
Aggregated score	3.0 ± 0.2^b^	2.2 ± 0.1^bc^	3.1 ± 0.1^c^
Food tasted	3.1 ± 0.2	2.7 ± 0.1^a^	3.2 ± 0.1^a^
Food present	3.2 ± 0.2^a^	2.4 ± 0.2^ac^	3.5 ± 0.2^c^
Food available	2.5 ± 0.2^b^	1.5 ± 0.2^bc^	2.7 ± 0.2^c^

Data presented as mean ± SEM. *P* values adjusted for multiple testing using Bonferroni correction and adjusted for age and preoperative BMI. *TFEQ*, Three-Factor Eating Questionnaire; *DEBQ*, Dutch Eating Behavior Questionnaire; *FCQ-S*, Food Craving Questionnaire State; *FCQ-T-r*, Food Craving Questionnaire Trait-reduced; *PFS*, Power of Food Scale; *SWL*, suboptimal weight loss; *OWL*, optimal weight loss. Means with same superscript letters are significantly different. ^a^*P* < 0.05; ^b,c^*P* < 0.01

Liking and wanting for food in the different study groups are shown in Table 3. The OWL group presented with a lower implicit wanting for HFSA and HFSW food, in both fasting and fed states, compared to the control group (*P* < 0.05 for all). It also presented with higher implicit wanting for LFSA food, in both fasting and fed states, compared with the control group and in fed state also compared with the SWL group (*P* < 0.05 for both). The OWL group also had a lower explicit liking and wanting for HFSA food, both in the fasting and fed state, compared to the control group (*P* < 0.05 and *P* < 0.01) and for HFSW food compared with both the SWL (*P* < 0.05) and control (*P* < 0.01) groups.

**Table 3:** Liking and wanting for food in the different study groups in the fasting and fed states

Fasting					Fed	
	SWL (*n* = 24)	OWL (*n* = 24)	Control (*n* = 25)	SWL (*n* = 24)	OWL (*n* = 23)	Control (*n* = 25)
Implicit wanting						
* HFSA*	− 5.3 (− 22.1, 17.0)	− 1.8 (− 18.3, 14.0)^a^	9.1 (− 5.8, 21.6)^a^	− 3.2 (− 15.8, 12.7)	− 3.9 (− 15.9, 10.5)^a^	13.4 (− 2.3, 20.5)^a^
* LFSA*	2.2 (− 12.9, 28.6)	17.6 (7.0, 39.2)^a^	− 1.8 (25.4, 19.2) ^a^	3.9 (− 16.6, 24.7)^a^	27.4 (7.4, 41.4)^ab^	4.06 (− 20.2, 19.9)^b^
* HFSW*	− 30.6 (− 53.9, − 4.2)	− 43.0 (− 51.1, − 19.5)^c^	− 15.1 (− 43.3, 7.2)^c^	− 28.2 (− 52.2, − 8.0)	− 41.2 (− 51.2, − 34.1) ^a^	− 13.1 (− 47.3, − 5.4)^a^
* LFSW*	16.2 (1.5, 29.0)	18.0 (4.4, 35.5)	12.8 (− 2.7, 20.1)	23.4 (8.7, 35.1)	21.9 (11.4, 38.1)	11.8 (0.04, 22.8)
Explicit liking						
* HFSA*	42.8 (17.1, 60.0)	27.3 (18.8, 37.8)^a^	46.8 (22.6, 57.6)^a^	17.6 (2.4, 50.7)	12.5 (1.5, 31.0)^c^	43.8 (17.9, 61.1)^c^
* LFSA*	50.4 (38.7, 70.0)	57.8 (36.8, 65.0)	52.5 (31.5, 63.3)	21.1 (8.2, 56.9)	28.8 (1.3, 55.0)	40.0 (14.4, 55.3)
* HFSW*	22.8 (6.3, 38.4)^a^	7.5 (1.3, 19.3)^ac^	31.0 (8.4, 52.1)^c^	4.1 (1.1, 21.1)^a^	1.50 (1.0, 11.3)^c^	31.0 (3.5, 52.0)^ac^
* LFSW*	51.4 (43.4, 62.8)	50.5 (42.5, 56.3)	38.5 (29.4, 61.5)	26.0 (9.6, 48.1)	33.8 (8.5, 55.0)	38.0 (21.8, 61.4)
Explicit wanting						
* HFSA*	40.8 (11.1, 56.0)	25.8 (14.8, 37.8)^a^	46.50 (24.6, 57.4)^a^	18.3 (2.6, 41.3)	12.3 (1.0, 26.5)^c^	37.8 (16.8, 49.9)^c^
* LFSA*	48.4 (39.7, 72.1)	56.00 (36.3, 65.5)	51.25 (32.1, 63.0)	21.5 (10.5, 55.3)	32.8 (4.0, 58.0)	39.5 (14.6, 52.6)
* HFSW*	26.4 (6.6, 36.5)^c^	8.5 (1.3, 19.0)^cd^	29.8 (7.9, 52.1)^d^	5.3 (1.3, 19.1)^a^	1.5 (1.0, 8.3)^c^	26.0 (4.5, 42.9)^ac^
* LFSW*	51.1 (43.8, 63.4)	51.5 (46.5, 55.5)	38.3 (27.8, 60.3)	28.0 (6.6, 46.2)	36.8 (2.8, 54.3)	40.0 (24.8, 56.5)

Data presented as median (25, 75 percentiles). Means with same superscript letters are significantly different. ^a,b^*P* < 0.05; ^c,d^*P* < 0.01. *HFSA*, high-fat savory; *LFSA*, low-fat savory; *HFSW*, high-fat sweet; *LFSW*, low-fat sweet; *SWL*, suboptimal weight loss; *OWL*, optimal weight loss

### Correlation Analysis

A moderate inverse association was found between %TWL and %EWL (and positive association with %WR) and the following variables: disinhibition and hunger (TFEQ), emotional eating, diffuse emotions and clearly labelled emotions (DEBQ), frequency of cravings (FCQ-T-r), and food available, food present and aggregated score (PFS). Moreover, a weak inverse correlation was found between external eating from DEBQ and %EWL (see Table 4). Additionally, the higher the implicit wanting for LFSA in fasting, the larger the %EWL and %TWL. A weak inverse association was also found between explicit liking and wanting for HFSW food in the fasting state and %TWL and %EWL (see Supplementary table 1).

**Table 4:** Correlation between scores from questionnaires and %EWL, %TWL and %WR

	% EWL		%TWL		%WR	
	*r*	*P* values	*r*	*P* values	*r*	*P* values
TFEQ						
Dietary restraint	− 0.003	0.984	− 0.061	0.680	− 0.201	0.176
Disinhibition	* − 0.627*	< *0.001*	* − 0.622*	< *0.001*	*0.594*	< *0.001*
Hunger	* − 0.473*	*0.001*	* − 0.475*	*0.001*	0.267	0.070
DEBQ						
Emotional eating	* − 0.603*	< *0.001*	* − 0.623*	< *0.001*	*0.467*	*0.001*
Cognitive restrained eating	− 0.158	0.284	− 0.158	0.092	0.038	0.802
External eating	* − 0.374*	*0.009*	* − 0.353*	*0.014*	0.135	0.365
Diffuse emotions	* − 0.596*	< *0.001*	* − 0.606*	< *0.001*	*0.515*	< *0.001*
Clearly labelled emotions	* − 0.582*	< *0.001*	* − 0.606*	< *0.001*	*0.438*	*0.002*
FCQs						
State	− 0.251	0.114	− 0.262	0.098	0.115	0.478
Trait-reduced	* − 0.531*	< *0.001*	* − 0.531*	< *0.001*	*0.353*	*0.013*
PFS						
Food available	* − 0.575*	< *0.001*	* − 0.549*	< *0.001*	*0.440*	*0.002*
Food present	* − 0.439*	*0.002*	* − 0.386*	*0.007*	0.285	0.052
Food tasted	− 0.142	0.335	− 0.195	0.183	0.186	0.210
Aggregated score	* − 0.504*	< *0.001*	* − 0.487*	< *0.001*	*0.386*	*0.007*

*%EWL*, percent excess weight loss; *%TWL*, percent total weight loss

## Discussion

The present study aimed to compare eating behaviour traits, hedonic hunger, food preferences and reward between participants with SWL and OWL 13 years post-RYGB, in addition to a non-surgical group control, matched for pre-operative BMI. The results indicate a more dysfunctional eating behaviour in both the SWL and control groups compared with the OWL group. More specifically a higher disinhibition and hunger, emotional eating and its subcategories, external eating, a higher frequency of cravings and a higher hedonic hunger was seen in the SWL and control groups, compared with the OWL group. Higher scores in these eating behaviour variables were also associated with lower %EWL and %TWL, and a larger %WR. Interestingly, no differences were seen between the SWL and the control group regarding eating behaviour traits or hedonic hunger. The SWL group also presented with a higher explicit liking and wanting for HFSW foods compared with the OWL group. The OWL group had a lower explicit liking and wanting, and implicit wanting for HFSA and HFSW foods compared with control. In addition, the OWL group had a higher implicit wanting for LFSA food compared with controls, both in fasting and fed states, and compared with the SWL group in the fed state.

In the present study, a lower disinhibition and hunger were found in the OWL compared with the SWL group and these variables were inversely associated with %EWL. These findings are in line with those from Amundsen et al. who found a higher degree of disinhibition in those experiencing SWL 5 years after RYGB [16]. Another study by Konttinen et al. found that WL post-bariatric surgery was predicted by low levels of disinhibition and hunger, assessed with TFEQ, at different time points after surgery, and that those with lower levels shortly after surgery had greater WL after 10 years [49].

Higher levels of emotional eating were also found in the SWL group in the present analysis. This is in line with a recent study where higher levels of emotional eating were found to be associated with higher WR and less WL 4 years after RYGB [19]. However, Amundsen et al. found no differences in emotional eating between SWL and OWL groups 4 years post-surgery [16]. Another study found that women with a higher degree of emotional eating had, in fact, more successful WL 8 years post-surgery [50]. However, only 15% of the patients had RYGB. Therefore, differences in follow-up time may explain the divergent results.

Both the SWL and control groups presented with significantly higher frequency of cravings, compared with the OWL group. Food cravings, in particular preoccupations with food, are common in bariatric surgery candidates [51] and those with higher scores on the subscale ‘guilt from cravings’ have been shown to experience less WL post-bariatric surgery [52].

The present study showed that higher hedonic hunger was associated with less TWL and EWL and that the SWL group had significant higher scores than the OWL group in most categories. The domains ‘food available’ and ‘food present’ have been shown to be inversely associated with percentage excess BMI loss post-gastric bypass, in a cross-sectional analysis [23]. Moreover, Ullrich et al., using a longitudinal design, reported a marked reduction in hedonic hunger aggregated scores, as well as the subdomains ‘food available’ and ‘food tasted’ post-surgery, and absolute WL was inversely associated with ‘food tasted’ score [22]. Differences in study designs, methods of assessing eating behaviour and time between surgery and follow-up assessments could explain some of the differences among studies.

In the present analysis, implicit wanting and explicit liking and wanting for HFSA and HFSW food were lower, and implicit wanting for LFSA food higher, in the OWL group, compared with the SWL and/or control groups. Less liking for sweet foods has previously been shown to be associated with a larger WL post-bariatric surgery in women [53]. The majority of the evidence shows that RYGB reduces hedonic hunger and changes food preferences towards foods low in fat and sugar [22, 23, 27, 54, 55]. It is therefore possible that a portfolio of dysregulated eating behaviours leads to increased preference for high-fat food, putting some individuals at risk of overeating and SWL following bariatric surgery. On the other hand, Søndergaard et al. reported that bariatric surgery did not change food preferences, but that altered food preferences were predictive of WL [28]. However, food preferences were measured with an ad libitum buffet. Even though food preferences may predict WL, WL has also been shown to alter food preferences [56]. Further research is needed to ascertain the direction of causality.

This study presents with both strengths and limitations. First, participants had RYGB 13 years earlier, and, therefore, our findings are likely to represent long-term results. Second, the study had a control group, allowing for comparisons with a group that resembles pre-operative conditions. Third, the food pictures shown in the LFPQ were adapted to the Norwegian diet [33]. Lastly, TFEQ was developed for obesity research, and both the TFEQ and the PFS have been validated in individuals with obesity [41, 45, 46, 57]. The main limitation of this study is its cross-sectional design and, as such, no inference of causality can be done. Also, the sex distribution was skewed with very few men, preventing the generalisation of the results to the whole bariatric population. Even though we aimed to have a non-bariatric control group matched for the pre-surgical BMI of the bariatric groups, the control group had a significantly lower BMI compared with the SWL (but not the OWL) group. However, we adjusted for pre-operative BMI in our analysis, so this difference is unlikely to have affected the results. Finally, EWL % was used to define the SWL and OWL groups.

There are many possible mechanisms not discussed in this paper that can be associated with WL failure and WR post-bariatric surgery, namely gut microbiota [58]. Nevertheless, RYGB is thought to promote WL mainly by reducing appetite, likely due to exaggerated release of satiety hormones, particularly glucagon-like peptide Y (GLP-1), and to a lower extent also peptide YY (PYY) in the post-prandial period [59]. It has been proposed that SWL and WR following RYGB can be a result of a blunted release of satiety peptides, but results are inconsistent and long-term results are lacking [60, 61]. In addition, behavioural, dietary, psychological and medical factors can all play a role in long-term WL following bariatric surgery [11, 62]. The use of GLP-1 analogues, as a weight loss aid, in patients with SWL or WR post-bariatric surgery has started to be investigated with promising results [63].

Successful WL maintenance in the long-term remains the biggest challenge in obesity management, also after bariatric surgery [8, 9]. In the present study, the SWL group was more comparable to the non-surgical control group in most variables, suggesting that SWL could be a result of dysfunctional eating behaviours. However, due the cross-sectional nature of this study, it cannot be ascertained if this is a cause or a consequence of SWL and/or WR post-RYGB. If dysfunctional eating behaviours are found to be the cause of SWL and WR in longitudinal studies, then pre-operative screening for eating behaviour, food preferences and hedonic hunger should be recommended [64, 65], as well as long-term follow-ups aiming at improving these behaviours in order to ensure progressive WL and prevent weight regain [66].

In conclusion, SWL 13 years after RYGB is associated with dysfunctional eating behaviours, greater liking and wanting for high-fat food and greater hedonic hunger. Future longitudinal studies are needed to clarify the direction of causality.

## Supplementary Information

Below is the link to the electronic supplementary material.

Supplementary file1 (DOCX 18 KB)
