# Supplementary material for: Suboptimal Weight Loss 13 Years After Roux-en-Y Gastric Bypass: Is Hedonic Hunger, Eating Behaviour and Food Reward to Blame?
Source: Obes Surg. 2022 May 4;32(7):2263–71. doi: 10.1007/s11695-022-06075-z (PMC9276719; doi:10.1007/s11695-022-06075-z)
Supplement: Supplementary file 1 — Supplementary file1 (DOCX 18 KB) [file 11695_2022_6075_MOESM1_ESM.docx]

Table 1 Supplementary. Correlation between liking and wanting for food, %EWL, %TWL and %WR

| % EWL | | | %TWL | | %WR | |
| --- | --- | --- | --- | --- | --- | --- |
|  | **r** | **P values** | **r** | **P values** | **r** | **P values** |
| IW1 HFSA | -0.117 | 0.433 | -0.085 | 0.570 | 0.005 | 0.972 |
| IW1 LFSA | *0.355* | *0.014* | *0.360* | *0.013* | -0.091 | 0.545 |
| IW1 HFSW | -0.184 | 0.215 | -0.159 | 0.285 | 0.024 | 0.873 |
| IW1 LFSW | 0.102 | 0.496 | 0.035 | 0.814 | -0.111 | 0.459 |
| EL1 HFSA | -0.169 | 0.255 | -0.147 | 0.324 | 0.078 | 0.604 |
| EL1 LFSA | 0.112 | 0.453 | 0.127 | 0.395 | -0.137 | 0.358 |
| EL1 HFSW | *-0.319* | *0.029* | *-0.321* | *0.028* | 0.049 | 0.745 |
| EL1 LFSW | -0.108 | 0.469 | -0.141 | 0.346 | 0.074 | 0.621 |
| EW1 HFSA | -0.132 | 0.376 | -0.103 | 0.489 | 0.044 | 0.769 |
| EW1 LFSA | 0.089 | 0.554 | 0.101 | 0.500 | -0.115 | 0.442 |
| EW1 HFSW | *-0.323* | *0.027* | *-0.323* | *0.027* | 0.073 | 0.624 |
| EW1 LFSW | -0.070 | 0.642 | -0.090 | 0.550 | 0.001 | 0.994 |
| IW2 HFSA | -0.071 | 0.633 | 0.041 | 0.785 | -0.047 | 0.757 |
| IW2 LFSA | *0.435* | *0.002* | *0.451* | *0.001* | -0.192 | 0.201 |
| IW2 HFSW | -0.279 | 0.057 | -0.264 | 0.073 | -0.009 | 0.952 |
| IW2 LFSW | 0.119 | 0.427 | 0.053 | 0.724 | -0.018 | 0.906 |
| EL2 HFSA | -0.160 | 0.283 | -0.119 | 0.426 | 0.029 | 0.849 |
| EL2 LFSA | 0.031 | 0.837 | 0.059 | 0.694 | -0.100 | 0.507 |
| EL2 HFSW | -0.204 | 0.169 | -0.171 | 0.249 | 0.036 | 0.812 |
| EL2 LFSW | 0.027 | 0.856 | 0.027 | 0.856 | -0.095 | 0.531 |
| EW2 HFSA | -0.156 | 0.296 | -0.115 | 0.443 | 0.024 | 0.876 |
| EW2 LFSA | 0.065 | 0.663 | 0.099 | 0.510 | -0.100 | 0.510 |
| EW2 HFSW | -0.202 | 0.172 | -0.171 | 0.250 | 0.013 | 0.930 |
| EW LFSW | 0.029 | 0.847 | 0.031 | 0.830 | -0.080 | 0.599 |

*IW1: Implicit wanting fasting; EL1: Explicit liking fasting: EW2: explicit wanting fasting: IW2: Implicit wanting fed; EL2: Explicit liking fed: EW2: Explicit wanting 2; %EWL: Percent excess weight loss; %TWL: Percent total weight loss. Data reported as P-values and Spearman correlation (r).*
